# Supplementary material for: Randomized Controlled Trials on Renin Angiotensin Aldosterone System Inhibitors in Chronic Kidney Disease Stages 3–5: Are They Robust? A Fragility Index Analysis
Source: J Clin Med. 2022 Oct 20;11(20):6184. doi: 10.3390/jcm11206184 (PMC9605379; doi:10.3390/jcm11206184)
Supplement: Supplementary file 1 [file jcm-11-06184-s001.zip › Table S2.pdf]

Primary Outcome	Trial	CKD<br>FI (p value)	No CKD<br>FI (p value)	Total<br>FI (p value)
**All-cause mortality**	**ACEi-placebo**			
	SOLVD		4 (0.031)	14 (0.012)
**CV mortality**	HOPE	10 (0.003)	22 (0.003)	
**Major cardiovascular events**	**ACEi-placebo**			
	HOPE	2 (0.0038)	32 (0.002)	
	PROGRESS	117 (<0.001)	48 (<0.001)	
	SAVE	18 (<0.001)	6 (0.024)	
	**ARB-active control**			
	CREATE (CV events)	1 (0.048)		
	CREATE (hospitalization for angina)	2 (0.035)		
	CREATE (hospitalization for angina)	12 (<0.001)		
**Major cerebrovascular events**	**ACEi-placebo**			
	HOPE		22 (0.001)	
	PROGRESS	15 (0.003)	27 (<0.001)	
**New or worsening nephropathy**	**ARB-placebo**			
	RENAAL (doubling creatininemia)	12 (0.007)		
	RENAAL (ESRD)	1 (0.046)		
	**ACEi- active control**			
	Marin	4 (0.014)		
	**ARB-active control**			
	CASE-J	3 (0.004)		
**Composite (doubling Cr, ESRD, death)**	**ACEi-placebo**			
	Hou	7 (0.004)		
	**ARB-placebo**			
	TRANSCEND		1 (0.037)	
	RENAAL	4 (0.033)		
**Composite (MI, stroke, CV deaths)**	**ACEi-placebo**			
	HOPE		80 (<0.001)	
**Median (range)**		5.5 (1-117)	22 (1-80)	
