# Supplementary material for: Randomized Controlled Trials on Renin Angiotensin Aldosterone System Inhibitors in Chronic Kidney Disease Stages 3–5: Are They Robust? A Fragility Index Analysis
Source: J Clin Med. 2022 Oct 20;11(20):6184. doi: 10.3390/jcm11206184 (PMC9605379; doi:10.3390/jcm11206184)
Supplement: Supplementary file 1 [file jcm-11-06184-s001.zip › Table S3.pdf]

Secondary Outcome	Trial	CKD FI (p value)	No CKD FI (p value)	Total FI (p value)
**All-cause mortality**	**ACEi-placebo**			
	ADVANCE			4 (0.038)
	SAVE	6 (0.016)		
	HOPE	21(<0.001)		
**CV mortality**	**ACEi-placebo**			
	SOLVD		3 (0.036)	
	ADVANCE			16 (0.007)
**All-cause hospitalization**	**ACEi-placebo**			
	SOLVD		15 (0.007)	
	**ARBs-active control**			
	E-COST	1 (0.033)		
**CV hospitalization**	**ACEi-placebo**			
	SOLVD	4 (0.025)	17 (0.006)	
**Heart failure hospitalization**	**ACEi-placebo**			
	SOLVD	33 (<0.001)	28 (<0.001)	
	HOPE	4 (0.011)		
**Major CV events**	**ACEi-placebo**			
	PROGRESS		7 (0.012)	
	HOPE		36 (0.003)	
	**ACEi-active control**			
	ALLHAT		55 (<0.001)	
	**ARBs-active control**			
	IDNT	11 (0.003)		
**Major cerebrovascular**	**ACEi-active control**			

**events**			
	ALLHAT (>90)		8 (0.01)
	ALLHAT (89-60)		12 (0.011)
	**ARBs-active control**		
	COPE		1 (0.049)
**New or worsening nephropathy**	**ACEi-placebo**		
	ADVANCE	5 (0.002)	
**Median (range)**		5.5 (1-33)	13.5 (1-55)
