# Supplementary material for: Randomized Controlled Trials on Renin Angiotensin Aldosterone System Inhibitors in Chronic Kidney Disease Stages 3–5: Are They Robust? A Fragility Index Analysis
Source: J Clin Med. 2022 Oct 20;11(20):6184. doi: 10.3390/jcm11206184 (PMC9605379; doi:10.3390/jcm11206184)
Supplement: Supplementary file 1 [file jcm-11-06184-s001.zip › Table S4 091022 revised.pdf]

Table S4. Quality assessment for included trials.

Trial	Sequence generation	Allocation concealment	Blinding			Incomplete outcome data	Selective outcome reporting	Other source of bias
			participants	personnel	outcome assessors			
**ACE-Is *vs.* placebo**								
SOLVD 2013[21]	LOW	UNCLEAR	LOW	LOW	UNCLEAR	LOW	LOW	LOW
ADVANCE 2010[24]	LOW	LOW	LOW	LOW	LOW	LOW	LOW	LOW
PROGRESS 2007[20]	LOW	LOW	LOW	LOW	LOW	LOW	LOW	LOW
PEACE 2006[19]	LOW	LOW	LOW	LOW	LOW	LOW	LOW	LOW
Hou 2006[25]	LOW	LOW	LOW	LOW	LOW	LOW	LOW	LOW
SAVE 2004[18]	LOW	LOW	LOW	LOW	LOW	LOW	LOW	LOW
HOPE 2001[23]	LOW	LOW	LOW	LOW	LOW	LOW	LOW	LOW
AIPRI 1996[22]	LOW	LOW	LOW	LOW	LOW	LOW	UNCLEAR	UNCLEAR
**ARBs *vs.* placebo**								
ORIENT 2011[29]	LOW	LOW	LOW	LOW	LOW	LOW	LOW	LOW
TRANSCEND 2011 [16]	LOW	LOW	LOW	LOW	LOW	LOW	LOW	LOW
RENAAL 2001[30]	LOW	LOW	LOW	LOW	LOW	LOW	LOW	LOW
IDNT 2003/2001[32]	LOW	LOW	LOW	LOW	LOW	LOW	LOW	LOW
**ACE-Is vs. active control**								
MacGregor 2005[14]	LOW	LOW	HIGH	HIGH	HIGH	LOW	LOW	LOW
Marin 2001[17]	UNCLEAR	UNCLEAR	HIGH	HIGH	HIGH	LOW	LOW	LOW
Cinotti 2001[13]	UNCLEAR	UNCLEAR	HIGH	HIGH	HIGH	LOW	LOW	LOW

Zucchelli 1992[15]	UNCLEAR	UNCLEAR	HIGH	HIGH	HIGH	LOW	LOW	LOW
ALLHAT 2006[33]	LOW	LOW	LOW	LOW	LOW	LOW	LOW	LOW
**ARBs vs. active control**								
HIJ-CREATE 2010[26]	LOW	LOW	HIGH	HIGH	LOW	LOW	LOW	LOW
CASE-J 2009[27]	LOW	LOW	HIGH	HIGH	LOW	LOW	LOW	LOW
E-COST 2005[28]	LOW	LOW	HIGH	HIGH	LOW	LOW	LOW	LOW
IDNT 2003/2001[32]	LOW	LOW	LOW	LOW	LOW	LOW	LOW	LOW
COPE 2013[31]	LOW	LOW	HIGH	HIGH	LOW	LOW	LOW	LOW
**Study used with three arms**								
ALLHAT 2006[33]	LOW	LOW	LOW	LOW	LOW	LOW	LOW	LOW
COPE 2013[31]	LOW	LOW	HIGH	HIGH	LOW	LOW	LOW	LOW
IDNT 2003/2001[32]	LOW	LOW	LOW	LOW	LOW	LOW	LOW	LOW
**RASI *vs*. active control or ACE-I *vs*. no_treatment**								
Mimura 2008[34]	LOW	LOW	HIGH	HIGH	HIGH	LOW	LOW	LOW
